# Heart Disease Death Rates Among Blacks and Whites Aged ≥35 Years — United States, 1968–2015

**DOI:** 10.15585/mmwr.ss6705a1

**Published:** 2018-03-30

**Authors:** Miriam Van Dyke, Sophia Greer, Erika Odom, Linda Schieb, Adam Vaughan, Michael Kramer, Michele Casper

**Affiliations:** 1 Department of Epidemiology, Rollins School of Public Health, Emory University, Atlanta, Georgia; 2 Division for Heart Disease and Stroke Prevention, National Center for Chronic Disease Prevention and Health Promotion, CDC, Atlanta, Georgia

## Abstract

**Problem/Condition:**

Heart disease is the leading cause of death in the United States. In 2015, heart disease accounted for approximately 630,000 deaths, representing one in four deaths in the United States. Although heart disease death rates decreased 68% for the total population from 1968 to 2015, marked disparities in decreases exist by race and state.

**Period Covered:**

1968–2015.

**Description of System:**

The National Vital Statistics System (NVSS) data on deaths in the United States were abstracted for heart disease using diagnosis codes from the eighth, ninth, and tenth revisions of the *International Classification of Diseases* (ICD-8, ICD-9, and ICD-10) for 1968–2015. Population estimates were obtained from NVSS files. National and state-specific heart disease death rates for the total population and by race for adults aged ≥35 years were calculated for 1968–2015. National and state-specific black-white heart disease mortality ratios also were calculated. Death rates were age standardized to the 2000 U.S. standard population. Joinpoint regression was used to perform time trend analyses.

**Results:**

From 1968 to 2015, heart disease death rates decreased for the total U.S. population among adults aged ≥35 years, from 1,034.5 to 327.2 per 100,000 population, respectively, with variations in the magnitude of decreases by race and state. Rates decreased for the total population an average of 2.4% per year, with greater average decreases among whites (2.4% per year) than blacks (2.2% per year).

At the national level, heart disease death rates for blacks and whites were similar at the start of the study period (1968) but began to diverge in the late 1970s, when rates for blacks plateaued while rates for whites continued to decrease. Heart disease death rates among blacks remained higher than among whites for the remainder of the study period. Nationwide, the black-white ratio of heart disease death rates increased from 1.04 in 1968 to 1.21 in 2015, with large increases occurring during the 1970s and 1980s followed by small but steady increases until approximately 2005. Since 2005, modest decreases have occurred in the black-white ratio of heart disease death rates at the national level. The majority of states had increases in black-white mortality ratios from 1968 to 2015. The number of states with black-white mortality ratios >1 increased from 16 (40%) to 27 (67.5%).

**Interpretation:**

Although heart disease death rates decreased both for blacks and whites from 1968 to 2015, substantial differences in decreases were found by race and state. At the national level and in most states, blacks experienced smaller decreases in heart disease death rates than whites for the majority of the period. Overall, the black-white disparity in heart disease death rates increased from 1968 to 2005, with a modest decrease from 2005 to 2015.

**Public Health Action:**

Since 1968, substantial increases have occurred in black-white disparities of heart disease death rates in the United States at the national level and in many states. These increases appear to be due to faster decreases in heart disease death rates for whites than blacks, particularly from the late 1970s until the mid-2000s. Despite modest decreases in black-white disparities at the national level since 2005, in 2015, heart disease death rates were 21% higher among blacks than among whites. This study demonstrates the use of NVSS data to conduct surveillance of heart disease death rates by race and of black-white disparities in heart disease death rates. Continued surveillance of temporal trends in heart disease death rates by race can provide valuable information to policy makers and public health practitioners working to reduce heart disease death rates both for blacks and whites and disparities between blacks and whites.

## Introduction

Heart disease is the leading cause of death in the United States (*1*). In 2015, heart disease accounted for approximately 630,000 deaths, representing one in four deaths (*1*). Nationally, racial disparities in heart disease mortality have persisted since at least the 1980s (*2*) and have been documented as the leading contributor to differences between blacks and whites in life expectancy (*3*). The National Academy of Medicine (NAM), formerly known as the Institute of Medicine, and *Healthy People 2020* have both called for increased understanding of health disparities by race and geographic area (*4*,*5*). NAM has called for surveillance systems that can measure disparities in heart disease by race and by contextual factors such as place of residence (*4*). Documenting trends in heart disease death rates by race and state provides valuable information to policy makers and public health practitioners for promoting continued decreases both for blacks and whites, along with decreases in disparities between blacks and whites, in heart disease mortality.

Previous studies have largely focused on differences in heart disease death rates between blacks and whites nationally, not by state, and for a limited period (*6*–*9*). Although these studies indicate that disparities in heart disease mortality between blacks and whites persist, little is known about how these racial differences vary by state and over an extended period. Moreover, reports that documented trends in race-specific heart disease death rates by geographic area (*10*) often did not measure trends in racial disparities over time and by location. This study provides a historical perspective on black-white disparities in heart disease death rates nationally and by state in the United States from 1968 to 2015.

## Methods

### Data Source

CDC’s National Vital Statistics Surveillance System (NVSS) collects birth and death records of U.S. residents. Death records are classified by underlying cause and other contributing causes of death and include limited demographic data such as sex, age, race, and state of residence. Death records were obtained for U.S. decedents aged ≥35 years who died during 1968–2015. The study period began in 1968 because it was the first year that mortality data were coded according to the *International Classification of Diseases, Eighth Revision* (ICD-8), and the accuracy of microdata files could not be ascertained for the years 1959–1967 (*11*). This study was restricted to persons aged ≥35 years because the etiology for heart disease among younger persons often is different than that for older persons (*12*). Bridged-race postcensal estimates for 2011–2015, bridged-race intercensal estimates for 2000–2010, and intercensal and unbridged estimates for 1968–1999 from NVSS and the U.S. Bureau of the Census (*13**,**14*) were used for population estimates to calculate death rates per 100,000 population. Rates were age standardized using direct standardization to the 2000 U.S. standard population with six 10-year age groups.

### Case Definition and Classification

Heart disease deaths were defined as those with an underlying cause of death in the ICD category of diseases of the heart (e.g., coronary heart disease, heart failure, atrial fibrillation, and myocardial infarction) because this category has been the most inclusive and has had the most consistent definition of heart disease over time. The following ICD codes were used for the eighth, ninth, and tenth ICD revisions: ICD-8 (1968–1978): 390–398, 402, 404, and 410–429; ICD-9 (1979–1998): 390–398, 402, 404, and 410–429; and ICD-10 (1999–2015): I00–I09, I11, I13, and I20–I51 (Table 1). Revisions of the ICD with changes in coding rules can cause temporal discontinuities in cause-of-death trends. However, comparability ratios (which measure the extent of such discontinuities) for heart disease ICD codes are very close to 1 for the entire study period, indicating that changes in the ICD do not substantially affect the ability to compare heart disease death rates across ICD revisions (*15*–*17*).

**TABLE 1:** Heart disease diagnosis categories and related *International Classification of Diseases* codes

Diagnosis	ICD codes and years		
	ICD-8 (1968–1978)	ICD-9 (1979–1998)	ICD-10 (1999–2015)
**Diseases of the heart (heart disease)**	**390–398, 402, 404, 410–429**	**390–398, 402, 404, 410–429**	**I00–I09, I11, I13, I20–I51**
Acute rheumatic fever	390–392	390–392	I00–I02
Chronic rheumatic heart diseases	393–398	393–398	I05–I09
Hypertensive heart disease	402	402	I11
Hypertensive heart and renal disease	404	404	I13
Ischemic heart diseases	410–414	410–414	I20–I25
Acute and subsequent myocardial infarction	410	410	I21–I22
Pulmonary heart disease and diseases of pulmonary circulation	—	415–417	I26–I28
Other forms of heart disease	420–429	420–429	I30–I51
Atrial fibrillation and flutter	427.4	427.3	I48
Heart failure	427.0, 427.1	428	I50

**Abbreviations:** ICD = *International Classification of Diseases;* ICD-8 = eighth ICD revision; ICD-9 = ninth ICD revision*;* ICD-10 = tenth ICD revision.

### Analysis

Annual age-standardized heart disease death rates were calculated using statistical software at the national level, by state, and for the District of Columbia (DC) from 1968 to 2015. State-level heart disease death rates can be statistically unreliable when based on small numbers; therefore, heart disease death rates were not calculated for specific state-race groups with <20 deaths. Death rates for the total population included all racial/ethnic subgroups in the United States. Race-specific rates were calculated for blacks and whites only.

The ratio of black-white heart disease death rates was examined over time. To estimate the standard error of the ratio, the age-standardized rates were assumed to be normally distributed, and the variance of the ratio was computed using the delta method. Using the standard errors of the ratios, 95% confidence intervals (CIs) were calculated. Black-white mortality rate ratios with CIs that include 1 represent approximately equal rates of heart disease deaths among blacks and whites. Ratios with upper CIs <1 indicate lower heart disease death rates among blacks than whites, and ratios with lower CIs >1 indicate higher heart disease death rates among blacks than whites. A relative measure of disparity was used to estimate black-white disparities in heart disease mortality to standardize the change over time in disparities across states (*18*).

Joinpoint regression was used to model heart disease death rate trends and black-white heart disease mortality ratio trends over time. The program fits a model and uses permutation tests to determine statistically significant changes in temporal trends, identifying the joinpoint (i.e., point at which the slope of the trend line changes). For the total population and by race for each state and nationwide, the annual percentage change (APC) for each joinpoint trend segment and the average annual percentage change (AAPC) for the trend for the entire study period were calculated. Trends were modeled using a log linear model, and the modified Bayesian information criterion (*19*) was used to detect statistically significant changes in trends. Statistical significance was set at p<0.05. Calculated heart disease death rates were graphed by year for the total population and by race. Quartiles of heart disease death rates were created for the maps based on the distribution of rates for blacks and whites combined. Calculated black-white heart disease mortality ratios were graphed by year. Rates and ratios were mapped by state using ArcGIS 10.4.1.

## Results

### Heart Disease Death Rates by Race

Heart disease death rates were similar for blacks and whites at the beginning of the study period. During the study period, both blacks and whites experienced substantial decreases in heart disease death rates, from 1,071.6 to 396.0 per 100,000 for blacks and from 1,032.3 to 326.3 per 100,000 for whites. (Table 2). However, overall decreases in heart disease death rates, measured as the AAPC, were slower among blacks (AAPC = 2.2% per year) than whites (AAPC = 2.4% per year). Consequently, after 1975, heart disease death rates were consistently higher among blacks than whites.

**TABLE 2:** Heart disease death rates* and average annual percentage change among adults aged ≥35 years, by state — United States, 1968–2015

State/Area	Rate						Average annual percentage change		
	Blacks		Whites		Total		Blacks	Whites	Total
	1968	2015	1968	2015	1968	2015	1968–2015	1968–2015	1968–2015
Alabama	902.1	454.9	958.5	442.1	**938.1**	**442.4**	-1.5	-1.7	**-1.7**
Alaska	—^†^	—	958.8	281.4	**769.1**	**296.7**	—	-2.2	**-1.7**
Arizona	921.4	326.6	824.4	270.9	**806.4**	**268.8**	-2.1	-2.4	**-2.3**
Arkansas	917.4	515.6	960.0	423.0	**946.0**	**431.1**	-1.3	-1.7	**-1.6**
California	865.0	389.6	906.5	298.9	**898.3**	**282.8**	-1.9	-2.4	**-2.6**
Colorado	1,011.5	324.0	889.9	249.5	**889.6**	**249.1**	-2.6	-2.7	**-2.7**
Connecticut	1,113.7	296.3	1,000.0	289.0	**1,001.6**	**286.6**	-2.8	-2.6	**-2.6**
Delaware	1,560.1	332.5	1,160.8	324.3	**1,203.4**	**321.1**	-3.1	-2.8	**-2.9**
District of Columbia	1,036.0	478.9	966.0	198.1	**1,016.3**	**363.4**	-1.8	-3.4	**-2.3**
Florida	940.9	315.4	846.2	288.1	**857.1**	**289.8**	-2.1	-2.3	**-2.3**
Georgia	1,005.0	383.2	998.2	341.6	**996.5**	**348.9**	-1.9	-2.2	**-2.2**
Hawaii	—	—	828.4	281.5	**811.5**	**260.7**	—	-2.3	**-2.4**
Idaho	—	—	894.9	306.4	**890.9**	**303.8**	—	-2.3	**-2.3**
Illinois	1,341.5	433.6	1,207.2	324.7	**1,223.1**	**332.4**	-2.5	-2.8	**-2.8**
Indiana	1,124.9	401.8	1,038.7	350.5	**1,043.7**	**352.3**	-2.1	-2.3	**-2.3**
Iowa	1,013.6	356.3	935.0	312.6	**935.3**	**312.3**	-2.3	-2.3	**-2.3**
Kansas	955.3	343.8	912.6	307.0	**912.5**	**307.8**	-2.4	-2.3	**-2.3**
Kentucky	1,327.1	377.7	1,059.0	384.6	**1,076.0**	**382.1**	-2.7	-2.1	**-2.2**
Louisiana	1,227.0	491.6	1,109.6	383.7	**1,136.5**	**410.0**	-1.9	-2.2	**-2.1**
Maine	—	—	1,132.0	306.4	**1,129.8**	**305.7**	—	-2.8	**-2.8**
Maryland	1,266.6	375.9	1,140.8	319.5	**1,156.8**	**326.9**	-2.7	-2.7	**-2.6**
Massachusetts	1,039.8	213.5	1,066.5	277.5	**1,063.4**	**268.9**	-3.3	-2.8	**-3.0**
Michigan	1,065.8	510.4	1,059.1	372.6	**1,062.8**	**385.9**	-1.6	-2.2	**-2.1**
Minnesota	562.3	208.9	872.3	226.4	**868.9**	**226.5**	-2.9	-2.9	**-2.9**
Mississippi	1,032.9	505.8	1,003.4	445.2	**1,008.7**	**464.5**	-1.6	-2.1	**-2.0**
Missouri	1,000.6	433.9	986.8	380.4	**989.1**	**382.9**	-1.8	-2.2	**-2.2**
Montana	—	—	837.8	297.4	**832.5**	**303.5**	—	-2.3	**-2.2**
Nebraska	1,066.1	304.3	884.5	301.6	**886.1**	**300.4**	-2.7	-2.3	**-2.4**
Nevada	908.9	483.9	914.8	398.3	**913.2**	**387.8**	-1.3	-1.7	**-1.8**
New Hampshire	—	—	1,064.4	292.0	**1,061.5**	**289.4**	—	-2.6	**-2.6**
New Jersey	1,246.6	354.0	1,171.8	330.7	**1,179.3**	**323.5**	-2.6	-2.7	**-2.8**
New Mexico	577.7	364.6	768.1	281.8	**741.4**	**275.9**	-2.1	-2.3	**-2.2**
New York	1,112.5	377.4	1,187.3	359.8	**1,186.0**	**352.8**	-2.3	-2.6	**-2.6**
North Carolina	1,119.3	355.3	999.2	306.4	**1,020.1**	**314.6**	-2.3	-2.5	**-2.5**
North Dakota	—	—	871.8	267.5	**873.6**	**273.8**	—	-2.6	**-2.6**
Ohio	1,087.9	421.2	1,111.4	367.8	**1,111.2**	**371.1**	-2.1	-2.3	**-2.3**
Oklahoma	876.8	500.1	915.1	446.3	**900.9**	**453.3**	-1.4	-1.6	**-1.6**
Oregon	827.9	200.4	877.0	268.6	**873.7**	**264.8**	-3.0	-2.6	**-2.6**
Pennsylvania	1,062.8	400.1	1,200.3	342.4	**1,192.8**	**344.7**	-2.1	-2.6	**-2.6**
Rhode Island	1,601.7	219.0	1,156.6	315.8	**1,162.5**	**310.6**	-4.5	-2.7	**-2.8**
South Carolina	1,143.6	399.9	1,129.4	327.0	**1,126.6**	**341.9**	-2.3	-2.5	**-2.5**
South Dakota	—	—	881.5	285.1	**882.1**	**292.0**	—	-2.5	**-2.6**
Tennessee	1,108.7	462.7	1,002.8	394.6	**1,015.5**	**400.6**	-1.7	-2.1	**-2.0**
Texas	920.3	417.3	885.6	331.1	**887.2**	**332.9**	-1.7	-2.1	**-2.1**
Utah	—	—	862.0	300.5	**857.4**	**297.3**	—	-2.1	**-2.1**
Vermont	—	—	1,046.1	298.9	**1,044.3**	**296.9**	—	-2.6	**-2.6**
Virginia	1234.1	356.6	1,069.6	294.2	**1,096.3**	**298.0**	-2.6	-2.6	**-2.7**
Washington	1,016.2	262.6	976.1	275.0	**976.9**	**266.9**	-2.6	-2.7	**-2.8**
West Virginia	1,226.8	388.6	1,089.3	372.1	**1,092.7**	**371.1**	-2.7	-2.3	**-2.3**
Wisconsin	980.9	387.4	981.1	298.2	**981.0**	**302.9**	-1.7	-2.6	**-2.5**
Wyoming	—	—	980.0	312.2	**971.3**	**309.6**	—	-2.5	**-2.5**
**Total**	**1,071.6**	**396.0**	**1,032.3**	**326.3**	**1,034.5**	**327.2**	**-2.2**	**-2.4**	**-2.4**

* Per 100,000 population.

^†^ State-level heart disease death rates were not calculated in cases with <20 deaths in the state within a group (total population, blacks, or whites) because the rates are considered statistically unreliable.

**FIGURE 1:**
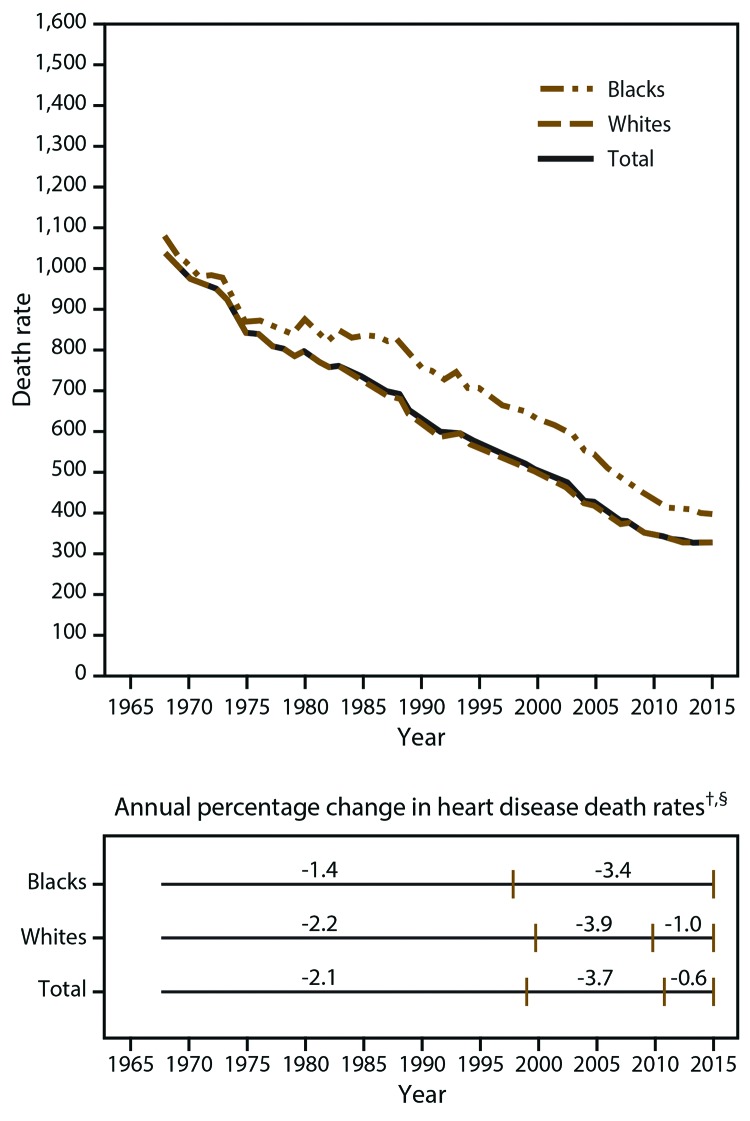
Heart disease death rates* and annual percentage changes among adults aged ≥35 years, by race — United States, 1968–2015 * Per 100,000 population, age standardized to the 2000 U.S. standard population. ^†^ Vertical lines indicate the year that the slope changed according to joinpoint trend analysis. ^§^ All annual percentage changes were significant (p<0.05), except -1.0 during 2010–2015 for whites and -0.6 during 2011–2015 for the total population.

Temporal patterns of decreases in heart disease death rates varied between blacks and whites. Among whites, a steady decrease in death rates occurred during 1968–2000 (APC = 2.2% per year), after which the decrease accelerated during 2000–2010 (APC = 3.9% per year) but slowed substantially during 2010–2015 (APC = 1.0% per year) (Figure 1). Among blacks, a slower decrease occurred than among whites during 1968–1998 (1.4% per year), although the decrease accelerated during 1998–2015 (APC = 3.4% per year). The trends (Figure 1) indicate that since approximately 2010, the decreases in heart disease death rates for blacks and whites have leveled.

**FIGURE 2:**
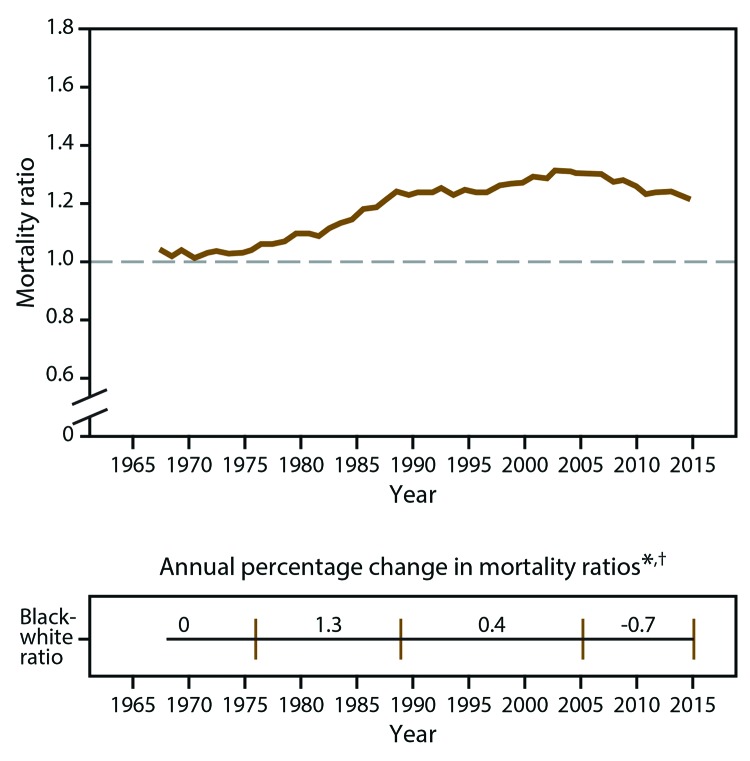
Black-white heart disease mortality ratios and annual percentage changes among adults aged ≥35 years — United States, 1968–2015 * Vertical lines indicate the year that the slope changed according to joinpoint trend analysis. ^†^ All annual percentage changes were significant (p<0.05), except 0.

Nationally, the black-white mortality ratio increased from the late 1970s to the mid-2000s (Figure 2). The black-white mortality ratio peaked in 2005 (1.31), followed by a modest decrease to 1.21 in 2015. The recent decrease in the black-white ratio reflects a larger rate decrease among blacks than whites during that period. Overall, the black-white disparity in heart disease death rates increased 16.3% from 1968 to 2015.

### Heart Disease Death Rates by Race and State

**FIGURE 3:**
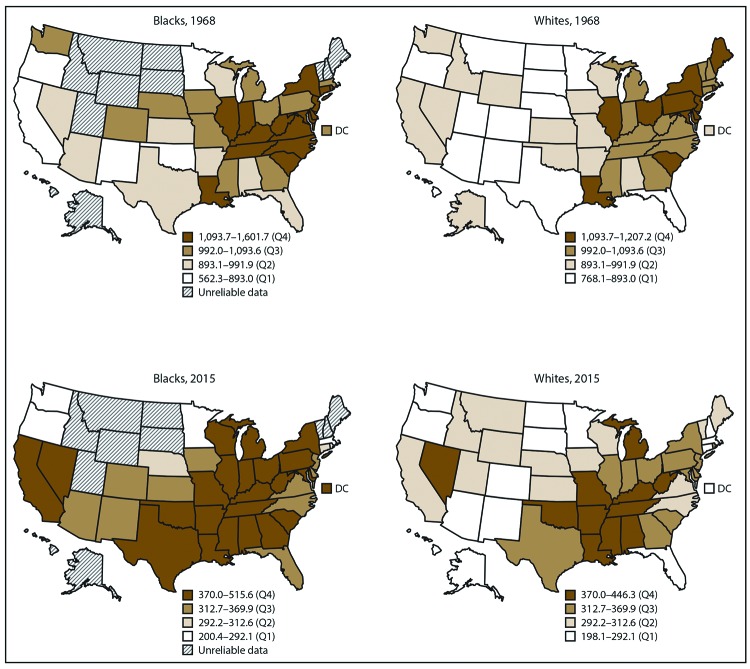
Heart disease death rates* among black and white adults aged ≥35 years — United States, 1968 and 2015 **Abbreviation:** Q = quartile. * Per 100,000 population, age standardized to the 2000 U.S. standard population. State-level heart disease death rates were not calculated when <20 deaths occurred in the state within a group (blacks or whites) because the rates are considered statistically unreliable. For each year, quartiles are based on the distribution of rates for blacks and whites combined. The use of consistent quartile cutpoints within each year allows for the comparison of the distribution of high and low rates for blacks vs. whites within 1968 and 2015, respectively.

Of the 51 geographic areas (50 states and DC), 39 states and DC had sufficient numbers of heart disease deaths among blacks to calculate statistically reliable heart disease death rates. All 51 geographic areas had sufficient numbers among whites. In 1968, heart disease death rates for blacks ranged from 562.3 (Minnesota) to 1,601.7 (Rhode Island) per 100,000 population and from 768.1 (New Mexico) to 1,207.2 (Illinois) for whites (Table 2). In 2015, heart disease death rates for blacks ranged from 200.4 (Oregon) to 515.6 (Arkansas) and for whites from 198.1 (DC) to 446.3 (Oklahoma) (Table 2). Both for blacks and whites, the geographic pattern of heart disease death rates changed over time. In 1968, the highest rates for blacks were concentrated primarily in the mid-Atlantic states, along with several midwestern and northeastern states, and the highest rates for whites were concentrated primarily in the Northeast and parts of the Midwest (Figure 3). In 2015, the highest rates for blacks were concentrated primarily in the northeastern, midwestern, and southern states, and the highest rates for whites were concentrated primarily in the south-central states. Comparison of U.S. maps showing heart disease death rates among blacks and whites in 2015 indicates that among blacks, a total of 23 of the state rates were in the highest quartile (based on the joint distribution of rates for blacks and whites), whereas among whites, a total of 11 of the state rates were in the highest quartile.

During 1968–2015, the AAPC in heart disease death rates varied by state both for blacks and whites (Table 2). Among blacks, the AAPC ranged from −4.5 per year (Rhode Island) to −1.3 per year (Arkansas and Nevada). Among whites, the AAPC ranged from −3.4 per year (DC) to −1.6 per year (Oklahoma). For the total population, the AAPC ranged from −3.0 per year (Massachusetts) to −1.6 per year (Arkansas and Oklahoma). State-specific graphs showing temporal trends in heart disease mortality by race and the black-white mortality ratio are available (Supplementary Figures, https://stacks.cdc.gov/view/cdc/51537). Trends in heart disease death rates for blacks and whites and black-white mortality ratios varied by state.

The black-white patterns for some states were similar to those at the national level; heart disease death rates for blacks were the same as for whites in 1968 but then diverged, with blacks having slower decreases and higher rates than whites for the remainder of the study period. For most states, the black-white mortality ratio increased from 1968 to 2015; however, in Massachusetts and Rhode Island, the ratio decreased; in some states, little change occurred. In 1968, black-white mortality ratios ranged from 0.64 in Minnesota to 1.38 in Rhode Island, and in 2015 they ranged from 0.69 in Rhode Island to 2.42 in DC (Table 3).

**TABLE 3:** Black-white ratios of heart disease death rates among adults aged ≥35 years, by state — United States, 1968 and 2015

State/Area	Black-white ratio (95% CI)	
	1968	2015
Alabama	0.94* (0.90–0.98)	1.03 (0.99–1.07)
Alaska	—^†^	—
Arizona	1.12 (0.95–1.32)	1.21* (1.10–1.32)
Arkansas	0.96 (0.90–1.02)	1.22* (1.14–1.30)
California	0.95* (0.92–0.99)	1.30* (1.27–1.34)
Colorado	1.14 (0.98–1.32)	1.30* (1.17–1.44)
Connecticut	1.11* (1.02–1.21)	1.03 (0.94–1.12)
Delaware	1.34* (1.21–1.50)	1.03 (0.91–1.15)
District of Columbia	1.07 (1.00–1.15)	2.42* (2.11–2.78)
Florida	1.11* (1.07–1.15)	1.09* (1.06–1.13)
Georgia	1.01 (0.97–1.04)	1.12* (1.09–1.16)
Hawaii	—	—
Idaho	—	—
Illinois	1.11* (1.08–1.14)	1.34* (1.29–1.38)
Indiana	1.08* (1.02–1.15)	1.15* (1.08–1.22)
Iowa	1.08 (0.89–1.32)	1.14 (0.97–1.34)
Kansas	1.05 (0.94–1.17)	1.12 (0.99–1.26)
Kentucky	1.25* (1.18–1.33)	0.98 (0.91–1.06)
Louisiana	1.11* (1.07–1.15)	1.28* (1.23–1.34)
Maine	—	—
Maryland	1.11* (1.07–1.16)	1.18* (1.13–1.23)
Massachusetts	0.98 (0.89–1.06)	0.77* (0.71–0.84)
Michigan	1.01 (0.97–1.04)	1.37* (1.32–1.42)
Minnesota	0.64* (0.50–0.84)	0.92 (0.82–1.04)
Mississippi	1.03 (0.98–1.08)	1.14* (1.08–1.19)
Missouri	1.01 (0.97–1.07)	1.14* (1.08–1.21)
Montana	—	—
Nebraska	1.21* (1.01–1.44)	1.01 (0.84–1.21)
Nevada	0.99 (0.71–1.39)	1.21* (1.12–1.32)
New Hampshire	—	—
New Jersey	1.06* (1.03–1.11)	1.07* (1.03–1.12)
New Mexico	0.75 (0.53–1.07)	1.29* (1.05–1.59)
New York	0.94* (0.92–0.96)	1.05* (1.02–1.08)
North Carolina	1.12* (1.08–1.16)	1.16* (1.12–1.20)
North Dakota	—	—
Ohio	0.98 (0.95–1.01)	1.15* (1.10–1.19)
Oklahoma	0.96 (0.88–1.05)	1.12* (1.04–1.21)
Oregon	0.94 (0.74–1.20)	0.75* (0.60–0.93)
Pennsylvania	0.89* (0.86–0.91)	1.17* (1.12–1.22)
Rhode Island	1.38* (1.15–1.66)	0.69* (0.56–0.86)
South Carolina	1.01 (0.97–1.06)	1.22* (1.17–1.28)
South Dakota	—	—
Tennessee	1.11* (1.06–1.15)	1.17* (1.12–1.22)
Texas	1.04* (1.01–1.07)	1.26* (1.23–1.29)
Utah	—	—
Vermont	—	—
Virginia	1.15* (1.11–1.20)	1.21* (1.16–1.26)
Washington	1.04 (0.91–1.19)	0.96 (0.86–1.06)
West Virginia	1.13* (1.02–1.25)	1.04 (0.88–1.24)
Wisconsin	1.0 (0.89–1.12)	1.30* (1.20–1.41)
Wyoming	—	—
**Total**	1.04* (1.03–1.05)	1.21* (1.21–1.22)

* Statistically significant. (95% confidence intervals do not include null value of 1.0.)

**^†^** State-level heart disease death rates were not calculated in cases with <20 deaths in the state within a group (blacks or whites) because those rates are considered statistically unreliable.

**FIGURE 4:**
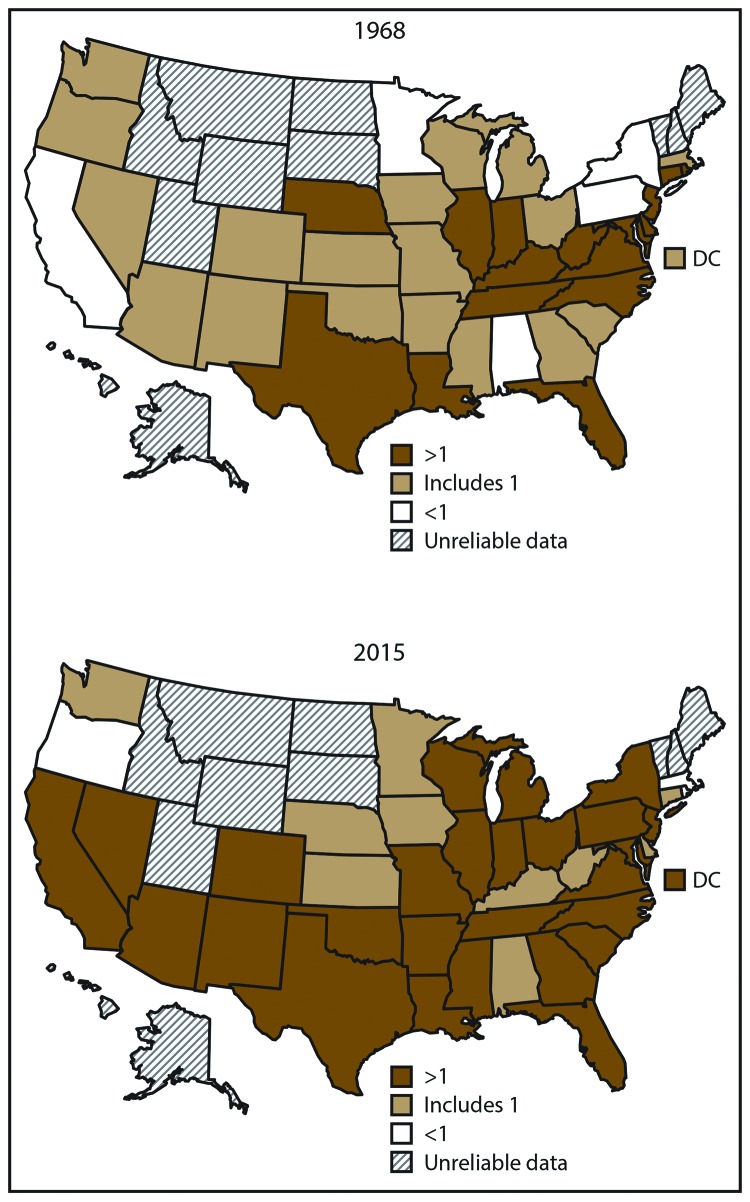
Black-white heart disease mortality ratios* among adults aged ≥35 years — United States, 1968 and 2015 * State-level heart disease death rates were not calculated when <20 deaths occurred in the state within a group (blacks or whites) because the rates are considered statistically unreliable. Ratio categories are based on statistical testing using 95% confidence intervals (CIs). Black-white mortality rate ratios with CIs that include 1 represent approximately equal rates of heart disease deaths among blacks and whites. Ratios with upper CIs <1 indicate lower heart disease death rates among blacks compared with whites, and ratios with lower CIs >1 indicate higher heart disease death rates among blacks compared with whites.

In 1968, 48% of the states (19 states) had black-white mortality ratios with CIs that included 1, indicating that distinguishing between black and white death rates in those states is not statistically possible (Figure 4). In 1968, approximately 13% of the states (five states) had black-white ratios <1, and approximately 40% of the states (16 states) had black-white ratios >1. By 2015, black-white mortality ratios had increased or remained unchanged for most states, resulting in 68% of the states (27 states) having black-white mortality ratios >1. Only 25% of the states (10 states) had ratios with CIs that included 1, and 8% of the states (three states) had black-white ratios <1. Overall, from 1968 to 2015, the number of states with black-white mortality ratios that were >1 increased from 16 (40%) to 27 (68%). In 1968, states with black-white heart disease mortality ratios that were >1 were concentrated primarily in the Midwest to the mid-Atlantic and Texas, Louisiana, Florida, and Nebraska, whereas in 2015, states with black-white ratios >1 were in parts of the Northeast, much of the Midwest and South, and parts of the West.

## Discussion

This report documents historical trends in black-white disparities in heart disease mortality, along with the total and race-specific trends in heart disease death rates, at both the national and state levels from 1968 to 2015. Nationally, although heart disease death rates decreased for the total population, the patterns of decrease differed by race and state. Heart disease death rates among blacks and whites decreased at comparable rates during the early portion of the study (1968 until the late 1970s) but then diverged from the late 1970s until the mid-2000s. During this time, whites experienced steady decreases in heart disease death rates. Blacks also experienced decreases; however, the rate of decrease was consistently slower than that for whites. This produced an increase in the black-white heart disease mortality ratio of 26% from 1.04 in 1976 to a peak of 1.31 in 2005, which was followed by a modest decrease in the black-white ratio of 7.6% to 1.21 in 2015. Overall, the black-white heart disease mortality ratio increased 16% from 1968 to 2015. At the state level, the majority of states experienced diverging trends in heart disease death rates by race and increases in black-white mortality ratios from 1968 to 2015; however, variations occurred among the states (Supplementary Figures, https://stacks.cdc.gov/view/cdc/51537) in the direction and magnitude of black-white disparities over time.

The decrease in heart disease death rates observed in this report for the total U.S. population has been well documented (*2*). From 1950 to 1996, heart disease death rates decreased 56% in the United States (*20*). Differences in heart disease mortality decreases by race have not been well documented at the state level, although several studies have documented spatiotemporal trends in heart disease mortality by race at the county level (*6*,*8*,*10*). A report documenting national trends in ischemic heart disease death rates for blacks and whites from 1981 to 1995 found similar racial disparities in the rates of decrease for ischemic heart disease death rates (*6*).

Factors contributing to national decreases in heart disease mortality in the United States are estimated to be approximately equally attributable to prevention and advances in treatment (*21*,*22*). From 1980 to 2000, approximately half of the reduction of coronary heart disease deaths in the United States was reported to be attributed to improvements in medical treatment, such as antihypertensive medications, cholesterol-lowering drugs, and revascularization for chronic angina (*21*). The other 50% of the decrease in heart disease deaths was reported to be a function of improvements in risk factors related to heart disease (e.g., reductions in cigarette smoking, hypertension, hyperlipidemia, and physical inactivity) (*20*,*21*). Black-white differences in the magnitude and patterns of decrease in heart disease death rates observed in this report suggest that blacks have not benefitted equally from the improvements in prevention and treatment that have contributed to the overall decreases in heart disease deaths in the United States. Specifically, differential or delayed access to or adoption of heart disease prevention and treatment across time among blacks compared with whites could be contributing to the observed increases in black-white disparities (*23*). In recent years, the modest improvements in the black-white heart disease mortality ratio coincide with a leveling off of decreases both for blacks and whites. This leveling might result from possible slowed progression in the favorable trends of heart disease prevention or treatment along with increases in the prevalence of certain risk factors (e.g., obesity) (*21*) both for blacks and whites. Additional research is needed to examine the conditions contributing to these patterns.

Although research examining whether factors contributing to temporal decreases in heart disease death rates vary by race is limited, racial disparities in heart disease risk factors, prevention, and treatment have been documented. On average, when compared with whites, blacks have higher prevalences of cardiovascular risk factors such as obesity (*26*), diabetes (*27*), hypertension (*28*), physical inactivity (*25*), and short sleep duration (*24*). With respect to clinical treatment of heart disease, blacks have been less likely to receive certain cardiovascular interventions and procedures (e.g., angiography, percutaneous transluminal coronary angioplasty, coronary artery bypass grafting, and thrombolysis therapy) than whites with similar characteristics (*29*). In the United States, socioeconomic factors, such as income, education, and access to adequate health care insurance (*23*,*30*–*32*), and race-related factors, including discrimination (*33*,*34*) and racial residential segregation (*35*,*36*), have been documented as underlying causes of racial differences in heart disease prevention and treatment (*37*).

Because certain risk factors for adult heart disease are evident years before clinical manifestation, a life course perspective might increase understanding of temporal patterns of death rates and disparities (*38*). For example, childhood and adolescent obesity (*39*,*40*), smoking (*41*), and childhood family socioeconomic status (*42*) are each predictors of incident cardiovascular disease in adults. Moreover, racial differences in these risk factors might exist between generations. An age-period cohort study examining heart disease death rates among U.S. blacks and whites from 1973 to 2010 found markedly increased rates of heart disease deaths specifically among blacks in the cohort born during 1920–1960 (*8*). More research is needed to examine how racial differences in risk factors across the life course and generations affect racial disparities in heart disease prevention and treatment.

The comparison of geographic patterns of black-white disparities in heart disease death rates at the beginning and end of the study period demonstrates widespread increases in black-white disparity among the states. The number of states with black-white heart disease mortality ratios that were >1 increased from 16 (40%) in 1968 to 27 (67.5%) by 2015. Although the black-white heart disease mortality ratio increased from 1968 to 2015 for most states, in several states, including Connecticut, Delaware, Kentucky, Massachusetts, Nebraska, Oregon, Rhode Island, Washington, and West Virginia, the black-white ratio remained equal or became favorable for blacks, with blacks having similar or lower heart disease death rates than whites. Examination of the race-specific trends in these states (Supplementary Figures, https://stacks.cdc.gov/view/cdc/51537), along with the race-specific 1968 and 2015 maps, reveals various circumstances that might have contributed to these results, such as a slowdown in the rate of decrease in heart disease death rates among whites compared with blacks in some states or an increase in the rate of decrease among blacks compared with whites in other states. In addition, in certain instances, heart disease death rates are high both for blacks and whites, resulting in black-white ratios that are approximately 1 (e.g., South Carolina in 1968 and Alabama in 2015). For states with high heart disease death rates both for blacks and whites, state-level policies and social norms that affect access to and adoption of heart disease prevention and treatment both for blacks and whites are particularly important to consider.

## Limitations

The findings in this report are subject to several limitations. First, this report only examines race-specific patterns of heart disease death rates among blacks and whites (*28*). Patterns for Hispanics, American Indians/Alaska Natives, and Asians/Pacific Islanders are not included because these racial/ethnic categories were not consistently reported on death certificates for the entire study period (*43*). Second, statistically stable trends of heart disease death rates among blacks, along with trends in black-white ratios, could not be calculated in 11 states because of the small populations of blacks in those states. Even in states for which both black and white rates could be calculated, small populations in some states resulted in black-white rate ratios with large CIs and wide variations across years. In these cases, interpretations of trends should be made cautiously and include examination of annual trends in race-specific heart disease death rates (Supplementary Figures, https://stacks.cdc.gov/view/cdc/51537). Third, because of the focus on geographic stratification and the extended study period, small, age-stratified populations of blacks limited the ability to conduct this analysis by age group (*9*). Fourth, because heart disease mortality data in this report are from death certificates, differences in adherence to reporting procedures for cause of death could lead to the misclassification of cause of death. Potential misclassification of heart disease subtypes is minimized in this report given the use of the broad ICD category “diseases of the heart” (*44*,*45*). Fifth, the use of statistical testing (via 95% CIs) to categorize states with increased, equal, or decreased black-white ratios might limit the ability to identify states with meaningful black-white disparities that are not statistically significant because of insufficient statistical power (i.e., small populations of blacks or whites). Sixth, as with any study of long-term temporal trends, the use of bridged-race estimates for the years 2000 and later could introduce a bias in race-specific trends if those persons reassigned a race category through the race-bridging method had self-identified as a different race category in previous census data. Although the direction of the potential bias is unknown, the magnitude of its impact is likely minimal given the small percentage of the population reporting multiple races (*46*). In addition, the bridged-race estimates provide the best population estimates available for comparison over time. Finally, this report examines black-white disparities in heart disease mortality at the state level and therefore does not include substate geographic variations that might exist within each state.

## Conclusion

This report highlights important black-white disparities in heart disease death rates at the national and state level in the United States from 1968 to 2015. Although heart disease death rates decreased both for blacks and whites during the study period and modest improvements occurred recently in the black-white heart disease mortality ratio, black-white disparities are persistent and concerning. Future research and initiatives should focus on improving national and state-level black-white disparities related to heart disease, which supports *Healthy People 2020* and NAM goals. The Million Hearts initiative (http://millionhearts.hhs.gov/learn-prevent/index.html), launched in 2012, outlines proven strategies to prevent myocardial infarctions, strokes, and deaths from cardiovascular disease and includes specific initiatives focused on the needs within black communities (*47*). In addition to using evidence-based strategies, standard treatment protocols, and culturally relevant tools to promote heart-healthy living, ensuring that heart disease prevention and treatment efforts are designed to benefit all groups equally is important. Ongoing attention to eliminating racial disparities in social determinants of health also are important to address racial disparities in heart disease prevention and treatment. The elimination of racial disparities in heart disease death rates, along with continued decreases in heart disease death rates for all persons in the United States, is important for the overall state of health. The trends in black-white disparities in heart disease death rates observed in this report highlight the importance of continued surveillance of these trends at the national and state level.

## References

[1] CDC, National Center for Health Statistics. Health, United States, 2016: with chartbook on long-term trends in health: Hyattsville, MD: National Center for Health Statistics, CDC, US Department of Health and Human Services; 2017. https://www.cdc.gov/nchs/data/hus/hus16.pdf 28910066

[2] Cooper R, Cutler J, Desvigne-Nickens P, Trends and disparities in coronary heart disease, stroke, and other cardiovascular diseases in the United States: findings of the national conference on cardiovascular disease prevention. Circulation 2000;102:3137–47. 10.1161/01.CIR.102.25.3137 11120707

[3] Kochanek K, Arias E, Anderson R. How did cause of death contribute to racial differences in life expectancy in the United States in 2010? NCHS Data Brief, No. 125. Hyattsville, MD: National Center for Health Statistics; 2013. https://www.cdc.gov/nchs/data/databriefs/db125.pdf 24152376

[4] Institute of Medicine Committee on a National Surveillance System for Cardiovascular and Select Chronic Diseases. The National Academies Collection: reports funded by National Institutes of Health. A nationwide framework for surveillance of cardiovascular and chronic lung diseases. Washington, DC: National Academies Press, National Academy of Sciences; 2011.

[5] US Department of Health and Human Services. Disparities. In: Healthy people 2020. Washington, DC: US Department of Health and Human Services. https://www.healthypeople.gov/2020/about/foundation-health-measures/Disparities

[6] CDC. Trends in ischemic heart disease death rates for blacks and whites—United States, 1981–1995. MMWR Morb Mortal Wkly Rep 1998;47:945–9. 9832470

[7] Gillum RF, Mehari A, Curry B, Obisesan TO. Racial and geographic variation in coronary heart disease mortality trends. BMC Public Health 2012;12:410. 10.1186/1471-2458-12-410 22672746 PMC3532343

[8] Kramer MR, Valderrama AL, Casper ML. Decomposing black-white disparities in heart disease mortality in the United States, 1973–2010: an age-period-cohort analysis. Am J Epidemiol 2015;182:302–12. 10.1093/aje/kwv050 26199382 PMC4528952

[9] Cunningham TJ, Croft JB, Liu Y, Lu H, Eke PI, Giles WH. Vital signs: racial disparities in age-specific mortality among blacks or African Americans—United States, 1999–2015. MMWR Morb Mortal Wkly Rep 2017;66:444–56. 10.15585/mmwr.mm6617e1 28472021 PMC5687082

[10] Vaughan AS, Quick H, Pathak EB, Kramer MR, Casper M. Disparities in temporal and geographic patterns of declining heart disease mortality by race and sex in the United States, 1973–2010. J Am Heart Assoc 2015;4:e002567. 10.1161/JAHA.115.002567 26672077 PMC4845281

[11] National Bureau of Economic Research. Mortality data—vital statistics. NCHS’ multiple cause of death data, 1959–2014. Cambridge, MA: National Bureau of Economic Research. http://www.nber.org/data/vital-statistics-mortality-data-multiple-cause-of-death.html

[12] Rubin JB, Borden WB. Coronary heart disease in young adults. Curr Atheroscler Rep 2012;14:140–9. 10.1007/s11883-012-0226-3 22249950

[13] US Census Bureau. Estimates of the resident population of the United States for July 1, 1968–July 1, 1989, by year, state, single-year of age (0, 1, 2, ≥85 years), race, Hispanic origin, and sex. Washington, DC: US Census Bureau; 2015. https://www.census.gov/programs-surveys/popest/data/data-sets.html

[14] CDC. Bridged-race estimates of the resident population of the United States for July 1, 1990–July 1, 2015, by year, state, single-year of age (0, 1, 2, ≥85 years), bridged race, Hispanic origin, and sex. Hyattsville, MD: National Center for Health Statistics, CDC; 2015. https://www.cdc.gov/nchs/nvss/bridged_race.htm

[15] Anderson RN, Minino AM, Hoyert DL, Rosenberg HM. Comparability of cause of death between ICD-9 and ICD-10: preliminary estimates. National Vital Stat Rep 2001;49:1–32. 11381674

[16] Casper M, Kramer MR, Quick H, Schieb LJ, Vaughan AS, Greer S. Changes in the geographic patterns of heart disease mortality in the United States: 1973 to 2010. Circulation 2016;133:1171–80. 10.1161/CIRCULATIONAHA.115.018663 27002081 PMC4836838

[17] Klebba A, Scott J. Estimates of selected comparability ratios based on dual coding of 1976 death certificates by the eighth and ninth revisions of the International Classification of Diseases. Hyattsville, MD: National Center for Health Statistics; 1980. https://www.cdc.gov/nchs/data/mvsr/supp/mv28_11s.pdf

[18] Mackenbach JP, Martikainen P, Menvielle G, de Gelder R. The arithmetic of reducing relative and absolute inequalities in health: a theoretical analysis illustrated with European mortality data. J Epidemiol Community Health 2016;70:730–6. 10.1136/jech-2015-207018 26945094

[19] Zhang NR, Siegmund DO. A modified Bayes information criterion with applications to the analysis of comparative genomic hybridization data. Biometrics 2007;63:22–32. 10.1111/j.1541-0420.2006.00662.x 17447926

[20] CDC. Decline in deaths from heart disease and stroke—United States, 1900–1999. MMWR Morb Mortal Wkly Rep 1999;48:649–56. 10488780

[21] Ford ES, Ajani UA, Croft JB, Explaining the decrease in U.S. deaths from coronary disease, 1980–2000. N Engl J Med 2007;356:2388–98. 10.1056/NEJMsa053935 17554120

[22] Goldman L, Cook EF. The decline in ischemic heart disease mortality rates. An analysis of the comparative effects of medical interventions and changes in lifestyle. Ann Intern Med 1984;101:825–36. 10.7326/0003-4819-101-6-825 6388454

[23] Phelan JC, Link BG. Controlling disease and creating disparities: a fundamental cause perspective. J Gerontol B Psychol Sci Soc Sci 2005;60:27–33. 10.1093/geronb/60.Special_Issue_2.S27 16251587

[24] Cunningham TJ, Wheaton AG, Ford ES, Croft JB. Racial/ethnic disparities in self-reported short sleep duration among U.S.-born and foreign-born adults. Ethn Health 2016;21:628–38. 10.1080/13557858.2016.1179724 27150351 PMC5206750

[25] Crespo SC, Smit E, Andersen RE, Carter-Pokras O, Ainsworth BE. Race/ethnicity, social class and their relation to physical inactivity during leisure time: results from the Third National Health and Nutrition Examination Survey, 1988–1994. Am J Prev Med 2000;18:46–53. https://www.ncbi.nlm.nih.gov/pubmed/10808982 10808982 10.1016/s0749-3797(99)00105-1

[26] Wang Y, Beydoun MA. The obesity epidemic in the United States—gender, age, socioeconomic, racial/ethnic, and geographic characteristics: a systematic review and meta-regression analysis. Epidemiol Rev 2007;29:6–28. 10.1093/epirev/mxm007 17510091

[27] Cowie CC, Rust KF, Byrd-Holt DD, Prevalence of diabetes and high risk for diabetes using A1C criteria in the U.S. population in 1988–2006. Diabetes Care 2010;33:562–8. 10.2337/dc09-1524 20067953 PMC2827508

[28] Mensah GA, Mokdad AH, Ford ES, Greenlund KJ, Croft JB. State of disparities in cardiovascular health in the United States. Circulation 2005;111:1233–41. 10.1161/01.CIR.0000158136.76824.04 15769763

[29] Lillie-Blanton M, Maddox TM, Rushing O, Mensah GA. Disparities in cardiac care: rising to the challenge of Healthy People 2010. J Am Coll Cardiol 2004;44:503–8. 10.1016/j.jacc.2004.04.043 15358011

[30] Williams DR, Jackson PB. Social sources of racial disparities in health. Health Aff (Millwood) 2005;24:325–34. 10.1377/hlthaff.24.2.325 15757915

[31] Meyer PA, Penman-Aguilar A, Campbell VA, Graffunder C, O’Connor AE, Yoon PW. Conclusion and future directions. In: CDC health disparities and inequalities report—United States, 2013. MMWR Suppl 2013;62:184–6. 24264513

[32] Weinick RM, Zuvekas SH, Cohen JW. Racial and ethnic differences in access to and use of health care services, 1977 to 1996. Med Care Res Rev 2000;57(Suppl 1):36–54. 10.1177/1077558700057001S03 11092157

[33] Lewis TT, Williams DR, Tamene M, Clark CR. Self-reported experiences of discrimination and cardiovascular disease. Curr Cardiovasc Risk Rep 2014;8:365. 10.1007/s12170-013-0365-2 24729825 PMC3980947

[34] Institute of Medicine. Unequal treatment: confronting racial and ethnic disparities in healthcare. Washington, DC: National Academies Press; 2003. 25032386

[35] Greer S, Kramer MR, Cook-Smith JN, Casper ML. Metropolitan racial residential segregation and cardiovascular mortality: exploring pathways. J Urban Health 2014;91:499–509. 10.1007/s11524-013-9834-7 24154933 PMC4074321

[36] Williams DR, Collins C. Racial residential segregation: a fundamental cause of racial disparities in health. Public Health Rep 2001;116:404–16. 10.1016/S0033-3549(04)50068-7 12042604 PMC1497358

[37] Mensah GA. Eliminating disparities in cardiovascular health: six strategic imperatives and a framework for action. Circulation 2005;111:1332–6. 10.1161/01.CIR.0000158134.24860.91 15769777

[38] Ben-Shlomo Y, Kuh D. A life course approach to chronic disease epidemiology: conceptual models, empirical challenges and interdisciplinary perspectives. Int J Epidemiol 2002;31:285–93. 10.1093/intjepid/31.2.285 11980781

[39] Morrison JA, Friedman LA, Gray-McGuire C. Metabolic syndrome in childhood predicts adult cardiovascular disease 25 years later: the Princeton Lipid Research Clinics follow-up study. Pediatrics 2007;120:340–5. 10.1542/peds.2006-1699 17671060

[40] Allcock DM, Gardner MJ, Sowers JR. Relation between childhood obesity and adult cardiovascular risk. Int J Pediatr Endocrinol. Epub October 19, 2009. https://www.ncbi.nlm.nih.gov/pmc/articles/PMC2775687 10.1155/2009/108187 PMC2775687 19956748

[41] Celermajer DS, Ayer JG. Childhood risk factors for adult cardiovascular disease and primary prevention in childhood. Heart 2006;92:1701–6. 10.1136/hrt.2005.081760 17041125 PMC1861256

[42] Nandi A, Glymour MM, Kawachi I, VanderWeele TJ. Using marginal structural models to estimate the direct effect of adverse childhood social conditions on onset of heart disease, diabetes, and stroke. Epidemiology 2012;23:223–32. 10.1097/EDE.0b013e31824570bd 22317806 PMC3414366

[43] Arias E, Schauman WS, Eschbach K, Sorlie PD, Backlund E. The validity of race and Hispanic origin reporting on death certificates in the United States. Vital Health Stat 2 2008;(148):1–23. 19024798

[44] Lloyd-Jones DM, Martin DO, Larson MG, Levy D. Accuracy of death certificates for coding coronary heart disease as the cause of death. Ann Intern Med 1998;129:1020–6. 10.7326/0003-4819-129-12-199812150-00005 9867756

[45] Ives DG, Samuel P, Psaty BM, Kuller LH. Agreement between nosologist and cardiovascular health study review of deaths: implications of coding differences. J Am Geriatr Soc 2009;57:133–9. 10.1111/j.1532-5415.2008.02056.x 19016930 PMC2631612

[46] Ingram DD, Parker JD, Schenker N, United States Census 2000 population with bridged race categories. Vital Health Stat 2 2003;(135):1–55. 14556588

[47] US Department of Health and Human Services. Preventing 1 million heart attacks and strokes. Washington, DC: US Department of Health and Human Services; 2014. https://millionhearts.hhs.gov/learn-prevent/index.html

